# Facial Morphogenesis of the Earliest Europeans

**DOI:** 10.1371/journal.pone.0065199

**Published:** 2013-06-06

**Authors:** Rodrigo S. Lacruz, José María Bermúdez de Castro, María Martinón-Torres, Paul O’Higgins, Michael L. Paine, Eudald Carbonell, Juan Luis Arsuaga, Timothy G. Bromage

**Affiliations:** 1 Center for Craniofacial Molecular Biology, Ostrow School of Dentistry, and Department of Anthropology, University of Southern California, Los Angeles, California, United States of America; 2 Centro Nacional de Investigación sobre la Evolución Humana, Burgos, Spain; 3 Centre for Anatomical and Human Sciences, Hull York Medical School, University of York, York, United Kingdom; 4 Institut Català de Paleoecologia Humana i Evolució Social, Tarragona, Spain; 5 Universidad Complutense de Madrid-Instituto Carlos III (UCM-ISCIII), Centro de Investigación de la Evolución y Comportamiento Humanos, Madrid, Spain; 6 Departments of Biomaterials and Biomimetics and Basic Science and Craniofacial Biology, New York University College of Dentistry, New York, New York, United States of America; University of Wisconsin, United States of America

## Abstract

The modern human face differs from that of our early ancestors in that the facial profile is relatively retracted (orthognathic). This change in facial profile is associated with a characteristic spatial distribution of bone deposition and resorption: growth remodeling. For humans, surface resorption commonly dominates on anteriorly-facing areas of the subnasal region of the maxilla and mandible during development. We mapped the distribution of facial growth remodeling activities on the 900–800 ky maxilla ATD6-69 assigned to *H. antecessor*, and on the 1.5 My cranium KNM-WT 15000, part of an associated skeleton assigned to African *H. erectus*. We show that, as in *H. sapiens*, *H. antecessor* shows bone resorption over most of the subnasal region. This pattern contrasts with that seen in KNM-WT 15000 where evidence of bone deposition, not resorption, was identified. KNM-WT 15000 is similar to *Australopithecus* and the extant African apes in this localized area of bone deposition. These new data point to diversity of patterns of facial growth in fossil *Homo*. The similarities in facial growth in *H. antecessor* and *H. sapiens* suggest that one key developmental change responsible for the characteristic facial morphology of modern humans can be traced back at least to *H. antecessor*.

## Introduction

The region of the modern human mid-face including the area below the nose is retracted relative to the upper face when compared with living and fossil hominoids and hominins. This condition, referred to as orthognathy, is one of the defining or autapomorphic features of modern humans [1]. From childhood to adulthood, the normal development of the human face experiences bone surface deposition, but also manifests bone removal (resorption) in key areas such as on the maxilla. Bone resorption and bone deposition are important cell-mediated mechanisms that, in addition to displacement, contribute to the balanced growth and spatial distribution of the various facial bones [2]–[5]. Specific distributions of remodeling fields reflect distinctive patterns of adult facial anatomy [2]–[5]. In the human face, orthognathy is associated with localized areas of bone resorption during ontogeny [2]–[5]. The activity of the different types of cells involved in bone deposition (osteoblasts) or bone resorption (osteoclasts) creates characteristically different surface features on both the outer, periosteal, and internal, endosteal, bone surfaces. When osteoclasts are active on the bone surface, they secrete acid and enzymes that break down bone matrix [6] resulting in very characteristic anisotropic resorption bays called Howship's lacunae [7]–[9]. In contrast, areas of bone deposition by osteoblasts lack Howship's lacunae, and instead have more isotropic surfaces that often contain bundles of mineralized collagen fibrils [7]–[9]. By direct observation, most commonly using the scanning electron microscope (SEM), it is possible to identify which type of cell activity predominated over periosteal surfaces, and this evidence can be used to reconstruct the remodeling events associated with facial growth in different fossil hominin taxa. This information can then be used to reconstruct the developmental basis of the phenotypic differences among fossil hominin taxa [8]–[12].

**Figure 1:**
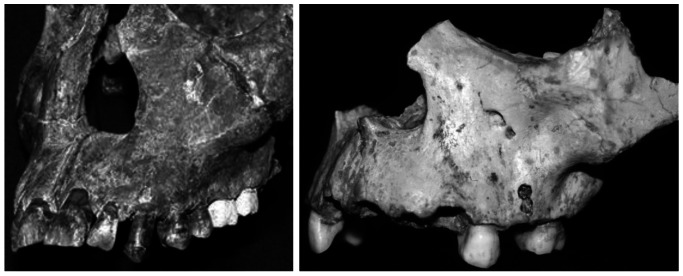
Lateral views of KNM-WT 15000 (left) and ATD6-69 (right). Note the differences in facial projection and in the topography of the maxilla.

The evolution of the genus *Homo* in Europe and its relationship with its African and Asian relatives remain important subjects of debate [13]–[19]. Here we characterize patterns of facial growth remodeling in two of the most complete sub-adult hominin facial skeletons recovered from the African and European Pleistocene relevant to understanding the evolution of the genus *Homo* [13], [17]–[19]. One of them, KNM-WT 15000, was originally assigned to African *H. erectus* and dates to ∼1.5 My [20]. The other specimen, ATD6-69, is assigned to *H. antecessor* and dates to 900–800 ky [14], [21]. The juvenile partial maxilla ATD6-69 was recovered from the sediments of the Gran Dolina site, Sierra de Atapuerca, Spain [14]. Interpretations of the morphological characteristics of ATD6-69 resulted in its classification as the novel species *H. antecessor,* and it remains the earliest evidence of a modern human-like, or orthognathic, mid-face [14], [15]. In contrast the geochronologically older *H. erectus* specimen KNM-WT 15000 (also known as the Nariokotome boy) from Kenya has a more projecting, or prognathic, mid-face than that of ATD6-69 (Fig. 1). This is one of a number of characteristics that warrant the two specimens being included in different species [14]–[15]. Some researchers have interpreted ATD6-69 as a late or a transitional form of *H. erectus* [18], [19]. Both ATD6-69 and KNM-WT 15000 preserve comparable anatomical regions of their facial skeletons and have a broadly similar dental age; in ATD6-69 the maxillary second molar (M^2^) is erupting whereas in KNM-WT 15000 this tooth is already erupted [14], [20]. The reasonably good preservation of these specimens and their significance in understanding the evolution of the genus *Homo*, prompted us to investigate their patterns of facial growth in the form of facial morphogenetic maps of remodeling activity. We reasoned that the differences in the degree of facial prognathism between ATD6-69 and KNM-WT 15000 could reflect different patterns of bone growth. If such differences exist, those differences would be consistent with the assignment of the two specimens to different taxa. Further, knowledge of these differences would provide new insights into the time when these important changes in facial growth took place.

### Interpreting Facial Growth Remodeling

Our knowledge of human facial surface growth and remodeling comes principally from studies of decalcified histological thin sections derived from formerly skeletonized specimens [2]–[4]. Interpretations of facial growth using this technique lack information concerning whether a developing surface was *actively* forming or resorbing at the time of death. Remodeling interpretations of such specimens were and continue to be based upon surface relief (in cross section) and underlying tissue organization, neither of which is able to provide any temporal resolution [7]. All that can be interpreted from such sections is what activity last characterized a particular surface, not whether the surface was actually active or not [2]–[4], [7]. To achieve temporal resolution would require attention to the presence and types of cells laying on the surface of histological sections taken from fresh specimens, the administration of vital bone labels just prior to death, and/or specific staining protocols that differentiate newly formed or resorbed bone from surfaces in which no activity was present.

Moreover, with the advent of scanning electron microscopy to analyze intact superficially anorganic bone preparations it was noticed that, in addition to actively forming and resorbing, bone surfaces could be classified as resting-forming and resting-resorbing [44]. Though this is a key feature of bone growth, the subtlety of this difference has not been exploited to determine the temporal intermittency of developing bone surfaces. Therefore, in the examination of dried or fossilized skulls, we can only say that the *last developing activity state* was either forming or resorbing.

Bone surfaces exhibit one activity state or the other i.e. forming, resorbing and resting. Despite the difficulty in interpreting such surfaces however, upon discovering a resorptive field that is commonly present we would describe the pattern as *typically* resorbing (vs resting resorbing) and develop a narrative that uses this information to describe how the bone grows [2]–[5], [7]–[11]. Further, while we know that surfaces may vary in their activity, they are not usually described as flip-flopping between forming and resorbing activities, except near to so called remodeling reversals (the “line” separating one activity from another) [7]. We might portray the subnasal clivus as, for example, resorbing in a modern human child only in so far as we repeatedly observe the traces of this activity, and it supports a narrative consistent with how we observe the face must have grown [4], [5]. Importantly, such a narrative works best when all of the surfaces are explained, including those of the mandible, to support a comprehensive interpretation of growth [7], [9]. Such interpretations are facilitated by our morphological descriptions. If there is *any* difference in morphology between two specimens within a species or between two specimens of any two species that is not simply due to extension or truncation of the same ontogenetic trajectory, then *by definition* there is a difference in growth remodeling between them. That difference can manifest as one either due to rates of activities, the pattern of forming or resorbing activities, or a combination of the two.

There is a known spatial variation in remodeling associated with developmental time as shown in cross sectional studies of modern skeletal materials [4], [5], [11]. The meaning of such variation bears intense scrutiny because it represents the sum of compensatory responses to various developing functional matrices. However, presently we do not have the tools to examine the source of this variation in detail and so presently our aim is to describe global phenomena. As an example, investigations of facial bone growth and remodeling in *Pan* have identified resorption over the clivus [11]. However, we know that the *average* trajectory of the chimpanzee maxilla is anteriorward [9], [11]. The question is then of how can these surfaces be resorbing posteriorward, and if they were, could this provide any grist for generalized interpretations of chimpanzee facial growth? At present we are only able to surmise that this resorptive surface must be a compensatory adjustment [9], [11], attributable perhaps to that particular stage of development [11], and one likely to reflect some transitory measure of downward growth. Thus, growth remodeling can be long lasting or transiently expressed and thus the key factor is the predominant bone state activity manifested through time that will more accurately explain facial growth. Alternatively, in the case of the fossil record where often juvenile fossil hominins are represented by single individuals such as in the present study, it is the differences in anatomy that are helpful in assessments of facial growth remodeling, as such they must reflect differences in activity state on bone surfaces.

## Materials and Methods

High resolution negative replicas of the fossils analyzed were obtained by RSL with permission from the Centro Nacional de Investigacion sobre la Evolución Humana (CENIEH) for ATD6-69, Burgos, Spain; and from the Nairobi National Museum, Kenya, for KNM-WT 15000. The preparation of the ATD6-69 specimen was carried out by the curators of the CENIEH, the repository for the *H. antecessor* fossils. Soluble preservative previously applied to the periosteal surface was removed by a soft brush and ethanol. Negative replicas were made as described [22] using Exaflex, an addition-cured light-bodied (injection type) silicone impression material (GC America Inc., Chicago, IL., U.S.A.). Positive replicas were prepared using Devcon 5-minute Epoxy (ITW Devcon, Danvers, MA). Uncoated positive replicas were examined by an EVO 50 scanning electron microscope (SEM) (Carl Zeiss, Thornwood, NY) in variable pressure secondary electron emission mode (15 kV accelerating voltage, 200 pA current, 8.5 mm working distance, 100 Pa pressure).

In addition, we used a portable confocal scanning optical microscope (PCSOM) [43] on ATD6-69. Osteoblast cells secrete an organic matrix that is subsequently mineralized and at intervals these cells become enclosed within the matrix in spaces called lacunae. These cells, called osteocytes, are connected to one another via protoplasmic processes, forming an interconnected system of cells called a syncitium. Moreover, when periosteal surfaces are forming, bone is typically laid down as a parallel-fibered tissue or arranged in sheet-like structures called lamellae. Osteocytes within these tissues are spatially well organized and have their long axes preferentially orientated with the principal orientation of the bone collagen [7]. In contrast, immediately below the continuously resorbing surfaces of bone of endosteal (or contralateral) origin, collagen orientation and, hence osteocytes, are not typically well organized [7]. It is therefore possible, in addition to SEM analysis of bone surfaces, to evaluate the remodeling activity state of a craniofacial periosteal bone by observing below-surface osteocyte lacunae distributions using confocal microscopy [7]. These sub-surface details are indeed a useful proxy for surface activities when the periosteal surfaces are damaged and preventing reliable interpretation of SEM images [7]. Images were acquired from the original specimen to depths of approximately 50 µm below the outermost surface. The relationships between osteocyte lacunae orientation and bone forming characteristics are illustrated in Figure S1.

Adherents on the facial skeleton of the Nariokotome boy specimen (KNM-WT 15000) were removed by RSL at the National Museums of Kenya. This procedure was carried out whilst periodically examining the specimen under a dissecting microscope to ensure that preservatives had been completely removed without damage to the specimen. Only selected areas of the specimen were cleaned. Areas where bone was thin or seemingly fragile and surfaces near glued joints were avoided. Small patches of the face and mandible were replicated as described above, labeled, and photographed for record keeping while the impression materials were still in place. The preservation of this specimen provided only a few areas where the predominant remodeling activity could be discerned, and only SEM of replicas has been performed to date. Therefore our interpretations of growth remodeling are less complete than those of ATD6-69. However, most areas of the nasomaxillary region were available for study in both specimens permitting direct comparisons (Fig. S2). Given the size of the KNM-WT 15000 cranium, it was impossible to examine its sub-periosteal facial surface with the PCSOM without ungluing parts of the skull, a curatorial procedure that was deemed problematic.

## Results

**Figure 2:**
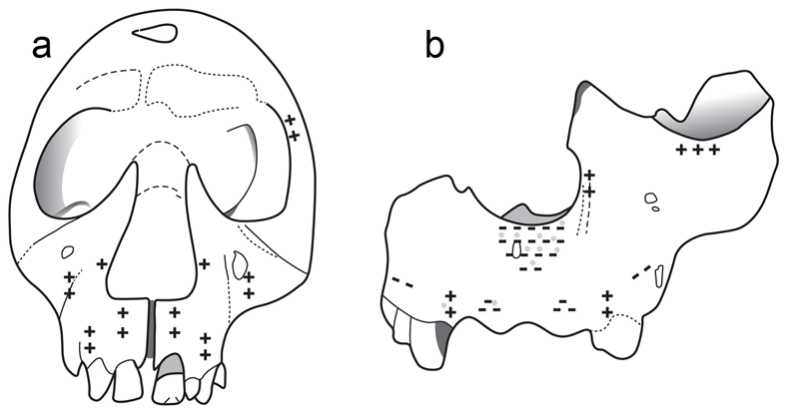
Facial growth remodelling maps. (**A**) Facial growth remodelling of the *H. erectus* specimen KNM-WT 15000 from Kenya, dating from ∼1.5 my showing depository fields (+) over most aspects of the anteriorly facing maxilla. Taphonomic alterations prevented a more complete analysis of the periosteal surface of this specimen which was only studied by SEM. (**B**) Facial growth remodelling of the specimen ATD6-69 representing *H. antecessor*, the oldest known European hominin species dating to 900–800 ky. SEM and confocal microscopy data showed resorptive fields (−) throughout the naso-alveolar clivus of this hominin, a characteristic shared with *H. sapiens*. Gray circles indicate the areas spot-mapped using the portable confocal microscope (PCSOM).

**Figure 3:**
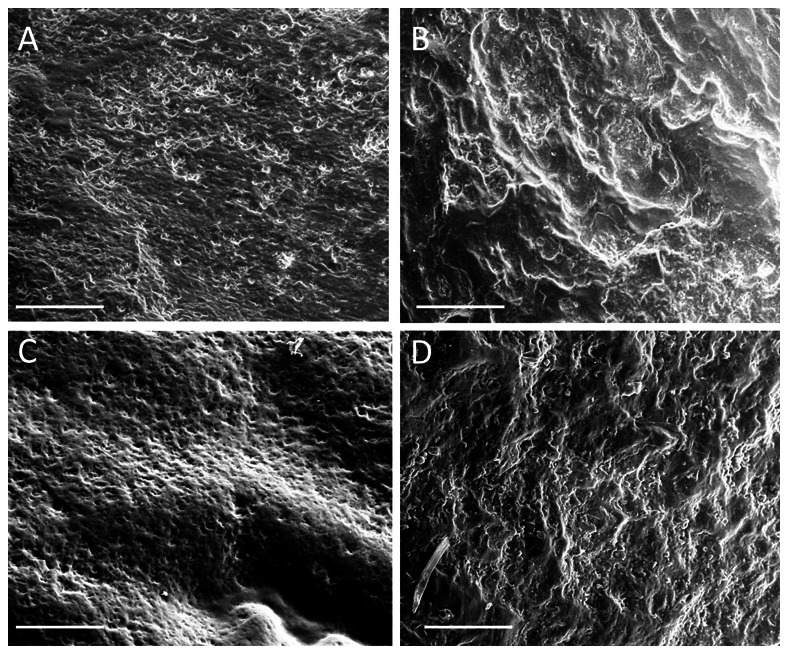
Scanning Electron Micrographs of facial growth remodeling in KNM-WT 15000 and ATD6-69. Images “**A”** and “**B”** are representative of growth remodeling fields in KNM-WT 15000 (*H. erectus*). Image “**A”** shows depository fields in the clivus area of this specimen. For comparison, “**B”** shows resorptive fields in the anterior aspect of the mandibular ramus of this specimen. Scale bars (A, B) = 50 µm. Images “**C**” and “**D**” represent growth remodeling fields of the specimen ATD6-69 (*H. antecessor*). Image “**C**” shows depository fields near the zygomatic region whereas “**D**” is a representative resorptive field in the clivus of ATD6-69. Scale bars (C,D) = 100 µm. All images shown here are taken from high resolution replicas examined in the scanning electron microscope.

Figure 2 shows the reconstructed facial morphogenetic maps for the two specimens; areas of net bone deposition are marked by (+) whereas areas of net bone resorption are indicated by the (−) symbol. Only the areas where we could confidently ascertain remodeling activity were marked by (+) or (−). Representative images of the surface micromorphology of areas bone resorption and deposition of the facial skeletons of ATD6-69 and KNM-WT 15000 are shown in Figure 3. Gray circles in Figure 2B indicate the areas spot-mapped using the PCSOM.

*KNM-WT 15000*: Figure 2A. *Naso-alveolar clivus*: The naso-alveolar clivus surface was largely depository at the time of death of KNM-WT 15000. *Nasal aperture and zygomatic:* The lateral walls of the nasal aperture were depository as was the right maxillary furrow. *Remaining maxilla*: The left side of the maxilla was characterized by depository fields from just above the alveolar bone of the I^2^ and dc. *Orbit*: The external surface of the left upper orbit was depository. *Mandible*: Resorption was evident on the anterior aspect of the ascending ramus of the mandible.

*ATD6-69*: Figure 2B shows the facial remodeling map of ATD6-69 in frontal view in which both SEM and PCSOM data have been combined (see Materials and Fig. S1). We have used images largely from the left side of the ATD6-69 maxilla, mirroring the corresponding remodeling characteristics to the right side. Choosing the left side of the maxilla was fortuitous as the right is less complete permitting us to more easily set the specimen on this side for PCSOM observation. *Naso-alveolar clivus*: The naso-alveolar clivus shows a predominantly resorptive pattern. *Nasal aperture and zygomatic*: The lateral walls of the nasal aperture and the anterior portion of the zygomatic were characterized by depository fields. *Remaining maxilla*: Aspects of the maxilla such as portions of the anterolateral maxilla and canine fossa showed resorptive characteristics, whereas islets of depository fields were identified over the canine prominence.

## Discussion

The facial bone growth remodeling map of KNM-WT 15000 (*H. erectus*) shows only depositional micromorphology on the naso-alveolar clivus, right nasal and right maxillary furrow. Clearly such deposition (Fig. 2A) can contribute to the anterior growth of the anterior maxilla and so to the differences in facial prognathism between KNM-WT 15000 and *H. sapiens.* Thus, the pattern of facial remodeling seen in this *H. erectus* specimen resembles the pattern seen in earlier hominins such as *H. habilis* and *Australopithecus* [7], [9], [11]. This pattern differs from members of the genus *Paranthropus* (*P. robustus* and *P. boisei*) which show fields of resorption on the subnasal region [7]–[11].

It should be noted that KNM-WT 15000 was originally attributed to *H. erectus* although some authors have proposed the use of the name *H. ergaster* for the early African *H. erectus* sample given the differences in morphology between early and late *Homo erectus*, particularly those from East Asia [23]. Despite this, others suggest that the observed variation between the African and Asian demes can be accommodated within a single species and that no significant differences can be credited to geographic variation [24]. While it is not the intention of this contribution to re-evaluate this potential taxonomic difference, it is important to highlight the known variation in the *H. erectus* face, both within early African and between African and Asian samples. For instance, the midfacial profile of KNM-WT 15000 is more pronounced than that of other early African specimens such as KNM-ER 3733 [24]. However, neither KNM-WT 15000 nor KNM-ER 3733 reveals a convex lower face in the transverse plane as is seen in some Asian specimens such as those in the Zhoukoudian or Sangiran samples [24]. In addition, KNM-WT 15000 shows no evidence of a canine fossa and its overall sub-nasal topography is flat [24]. In contrast, the lower face of ATD6-69 shows a convex sub-nasal region [14], [15] as in some Asian *H. erectus* specimens. It is appropriate to note here that an analysis of dental traits of Gran Dolina specimens suggests a stronger link with Asian samples than with early African *H. erectus* [25], [26]. It is therefore important to indicate that it is presently unknown whether the depository surfaces identified in this study on the KNM-WT 15000 face are representative of a developmental process characterizing *H. erectus* as a whole or simply reflect an early African *H. erectus/ergaster* status.

A key morphological feature bearing on the attribution of ATD6-69 to a novel taxon is its mid-facial retraction [13]. Strikingly, the reconstruction of the facial bone growth remodeling map of ATD6-69 shown in Figure 2B identifies bone resorption over the naso-alveolar clivus, as is common in *H. sapiens*. Histological studies of facial growth remodeling in humans were pioneered by Enlow [2]–[5]. This series of papers documented resorption as the predominant activity of the external aspect of the nasomaxillary complex and the subsequent facial maps provided in these studies depicted resorption uniformly distributed over the whole anterior lower face [2], [3]. A subsequent histological study [4] reported in more detail on the distribution of remodeling fields on the human face. This study revealed that although resorption is indeed the predominant activity, some variation in the extent of remodeling fields was evident [4]. In that study, 89% of the specimens with primary dentition erupted or with mixed dentition (n = 27) presented large resorptive areas over the clivus, but 100% showed resorptive fields over various parts of the maxilla [4]. Bromage’s [9] sample of six individuals with at least the first permanent molar erupted was studied using replicas of the face to map surface activity using the SEM. Results showed resorption over a portion of the clivus in 5 out of the 6 specimens. Using the same technique, McCollum [11] however found greater variation than previous studies had reported. In a sample of 22 *H. sapiens* individuals with either primary or permanent dentition erupted, 55% were reported to have some surface *deposition* along the nasoalveolar clivus [11]. Importantly however, resorption was found throughout many regions of the anterior lower face in this sample, consistent with resorption being a contributing factor to the orthognathic profile of *H. sapiens* relative to prognathic primates such as chimpanzees [2], [9], [11]. In this regard, McCollum [11] also found that *Pan troglodytes*, particularly in older individuals, showed some resorptive patches over the clivus which contrasted with Bromage’s study [9] wherein a portion of the clivus of all *P. troglodytes* individuals analyzed showed only depository fields. However, as discussed earlier, McCollum [11] noted that it was unlikely that the resorption observed in older *P. troglodytes* individuals greatly modifies the general forward growth vector of the chimpanzee face ([11], p. 12). Likewise, it can be surmised that, despite the variation in the extent of remodeling fields reported by these studies [4], [9], [11], resorption over the nasomaxillary complex remains a key factor contributing to the less pronounced forward growth of the modern human face. The similarity in the pattern of facial remodeling in ATD6-69 and *H. sapiens* is thus likely associated with a retracted subnasal region in comparison with the condition in African apes and in earlier hominins [2], [4], [9], [11].

With regard to the facial remodeling data presented here for two specimens, ATD6-69 and KNM-WT 15000, we cannot know the extent to which the remodeling features identified in these individuals are typical. However, given the important anatomical differences, particularly in the topography of the nasomaxillary complex and anterior projection of the lower face between ATD6-69 and KNM-WT 15000 (see Fig. 1), and the unique features of ATD6-69 face [18] more akin to *H. sapiens* [14], it is unlikely that the differences in remodeling identified in this study are completely unassociated with their distinct facial morphologies. As such they present new evidence that point toward differences in morphogenesis.

Key differences (proportioning) in cranial form among apes are present at birth [27], [28] a time when the bones of the face are characterized by deposition [2]–[5]. During postnatal growth the ape phenotypes diverge through a combination of differences in ontogenetic vector directions and magnitudes [2]–[5]. Thus, the initial geometry of muscles, teeth and jaws is set up during early development under various genetic signals that regulate craniofacial patterning and early growth, but that geometry can also be influenced by mechanical and spatial interactions. Masticatory function not only impacts the development of the craniofacial skeleton but it is also essential for normal growth and development of the skull [29]. Thus, facial bones respond to the genetic and local mechanical milieu through variations in the spatial and temporal interplay of depository and resorptive activity. Yet, it is well known that the cranium is highly morphogenetically integrated [27], [29]; with changes in one region having a wide impact. One reason is that morphological changes in one region can alter the strain that is occurring elsewhere [30] as result of e.g. masticatory sytem activity. Thus, a morphological change due to loading in one part of the cranium may impact on remodelling activity in another. How mechanical function impacts on cranial remodeling has been considered previously in an evolutionary context for facial growth remodeling. Thus, orthognathy, integrated within a suite of other cranial characteristics (e.g., cranial base flexion) [1], [31], has been linked with a more efficient (in the sense of greater bite force relative to muscle force) posterior bite [32] in *Paranthropus* relative to *Australopithecus.* Differences in anatomy and mechanical performance between the taxa in these genera are associated with reduced prognathism as well as increased post-canine tooth size, and greater mandibular robusticity and ramus height in *Paranthropus.* These architectural and functional differences may relate to the differences in growth remodeling of the mid-face in these taxa. Thus, the resorptive field over the maxilla in *Paranthropus* [9], [11] could plausibly arise as result of altered mechanical loading (and so mechanical signaling) relative to the condition in *Australopithecus* and contribute to the development of a more orthognathic face. The modern human-like mid-facial form and the modern human-like pattern of facial remodelling in *H. antecessor* is quite distinct from the hyper-robust trend displayed by *Paranthropus* and it is less clearly associated with such changes.

Thus, the similar pattern of subnasal remodelling and anatomy in modern humans and *H. antecessor* could be evidence of a shared mechanism for mid-facial retraction among more recent fossil hominins. The shared characteristics include the locations, extent and activity rates of resorptive fields; all are factors that would affect the degree of mid-facial retraction. Another potential factor accounting for differences in facial growth remodelling between species may be brain size. Brain development is a key determinant of mammalian craniofacial architecture [1], [32], [33]. An increase in brain size can cause anatomical readjustments (i.e., influence cranial flexion, re-orientate cranial musculature) that may also influence the vocal tract [1], [31]. Such changes in musculoskeletal architecture will lead to a change in cranial deformation arising from masticatory system loads, a process associated with ‘compensatory’ remodelling of the mid-face [4], [9]. The increase in cranial capacity from the 880 cm^3^ reported for KNM-WT 15000 [34], [35] to the endocranial volume in excess of 1000 cm^3^ that has been estimated for *H. antecessor* [36], by itself may result in differences in strain-related morphogenesis that could have elicited resorptive remodelling over the subnasal region and an alteration of growth trajectory between these two taxa. Whether this association stands when other species of the genus *Homo* are included from Africa and Eurasia; needs to be determined. A possible exception from the model proposed here may be the large-brained and somewhat more prognathic Neandertals, but any differences may be linked to the development of derived facial characteristics or other specializations described for this latter taxon [37]. These hypotheses regarding the regulation and specific variations in facial remodelling among hominins could be tested through finite elements analysis applied to artificially varied (manipulated, virtual) crania [38] and a more complete sampling of fossil *Homo*.

Other lines of evidence, in addition to facial growth remodelling, may contribute understanding the differences in presumed growth characteristics of *H. antecessor* and *H. erectus*. Dental development (eruption patterns and enamel crown development) is fully integrated within the growth of the craniofacial complex and can be used as a proxy for determining growth trajectories of species [9], [39], [40]. The growth pattern of *H. erectus* based on enamel microstructural details appears more akin to that of earlier hominins [39] and in particular that of KNM-WT 15000 [41]. In contrast dental eruption/mineralization patterns for *H. antecessor* suggest a more modern human dental developmental schedule for this species [42]. The identification of similar dental growth patterns in *H. sapiens* and *H. antecessor* together with similar growth mechanisms in the facial skeleton suggest that an important shift towards “modernization” in developmental characteristics were present in the genus *Homo* by 900–800 years ago.

### Conclusion

We have provided evidence that the *H. erectus* specimen KNM-WT 15000 has a pattern of facial growth remodeling that is similar to that seen in early *Homo* and *Australopithecus* and it is unlike the pattern seen in *H. antecessor* or *H. sapiens*. The similarities between the subnasal anatomy of *H. antecessor* and *H. sapiens* [13] and the shift to a predominance of resorption during later facial growth in *H. antecessor* suggests that at least one important element of the “modernization” of the face was clearly underway in *H. antecessor*.

## Supporting Information

Figure S1 **Identification of osteocyte orientation and bone forming characteristics.** When periosteal surfaces are forming, bone is typically laid down as a parallel-fibered tissue or arranged in sheet-like structures called lamellae. Bromage and Boyde (2008) [7] described that osteocytes within these tissues are spatially well organized and have their long axes preferentially orientated with the principal orientation of the bone collagen. In contrast, osteocytes beneath resorbing periosteal surfaces are not typically well organized. To illustrate this principle, we have analyzed histological bone section of rat mandibles. *Animal and Tissue Preparation*: All animal manipulation conformed to University and Federal Guidelines. Two ∼100 g rats were perfused intracardiacally with 4% paraformaldehyde (PFA), mandibles were dissected free and soft tissues removed. After additional overnight fixation of the mandibles in PFA, samples were decalcified for 3 weeks in 4% EDTA, washed in buffer, embedded in paraffin and sectioned. Sections were stained following standard Hematoxylin-Eosin protocols and cover slipped to be imaged by an Olympus BH2 microscope. Figure S1, Panel A, shows paralleled fibered lamellar bone tissue (small white arrows) and osteocytes (Ocy), the latter showing a predominant orientation following that of the lamellae. In contrast, Panel B shows poorly identifiable lamellae and osteocyte orientation is random relative to the field of view. This principle applies to the imaging of ATD6-69 using portable confocal microscope as described [43]. The resulting confocal images for bone resorption or deposition are shown at the bottom of Fig. 1S. The confocal image on the bottom of Panel A thus represents forming bone surfaces with osteocytes arranged in a paralleled fashion. This image was taken from ref [7]. The image at bottom of Panel B was obtained from the clivus area of ADT6-69 and shows haphazard orientation of osteocytes thus representing a resorptive surface. In both cases images were acquired from the original specimen to depths of approximately 50 µm below the outermost surface.
(TIF)

Figure S2 **KNM-WT 1500 during casting of key areas of the face.** KNM-WT 15000 during casting of key areas of the face. The facial skeleton of the Nariokotome boy specimen (KNM-WT 15000) was carefully prepared for study onsite at the National Museums of Kenya by one of us (RSL) by removing adherents. This procedure was carried out whilst periodically examining the specimen under a dissecting microscope to ensure that preservatives had been completely removed without damage to the specimen. Only selected areas of the specimen were cleaned. Areas where bone was thin or seemingly fragile and surfaces near glued areas were avoided. Small patches of the face and mandible were replicated as described above, labeled, and photographs taken for record keeping while the impression materials was still in place. Permission to study the specimen was kindly granted by the authorities of the National Museums of Kenya and supervised by Dr. Emma Mbua.
(TIF)
