# Supplementary figures and images for: Facial Morphogenesis of the Earliest Europeans
Source: PLoS One. 2013 Jun 6;8(6):e65199. doi: 10.1371/journal.pone.0065199 (PMC3675139; doi:10.1371/journal.pone.0065199)

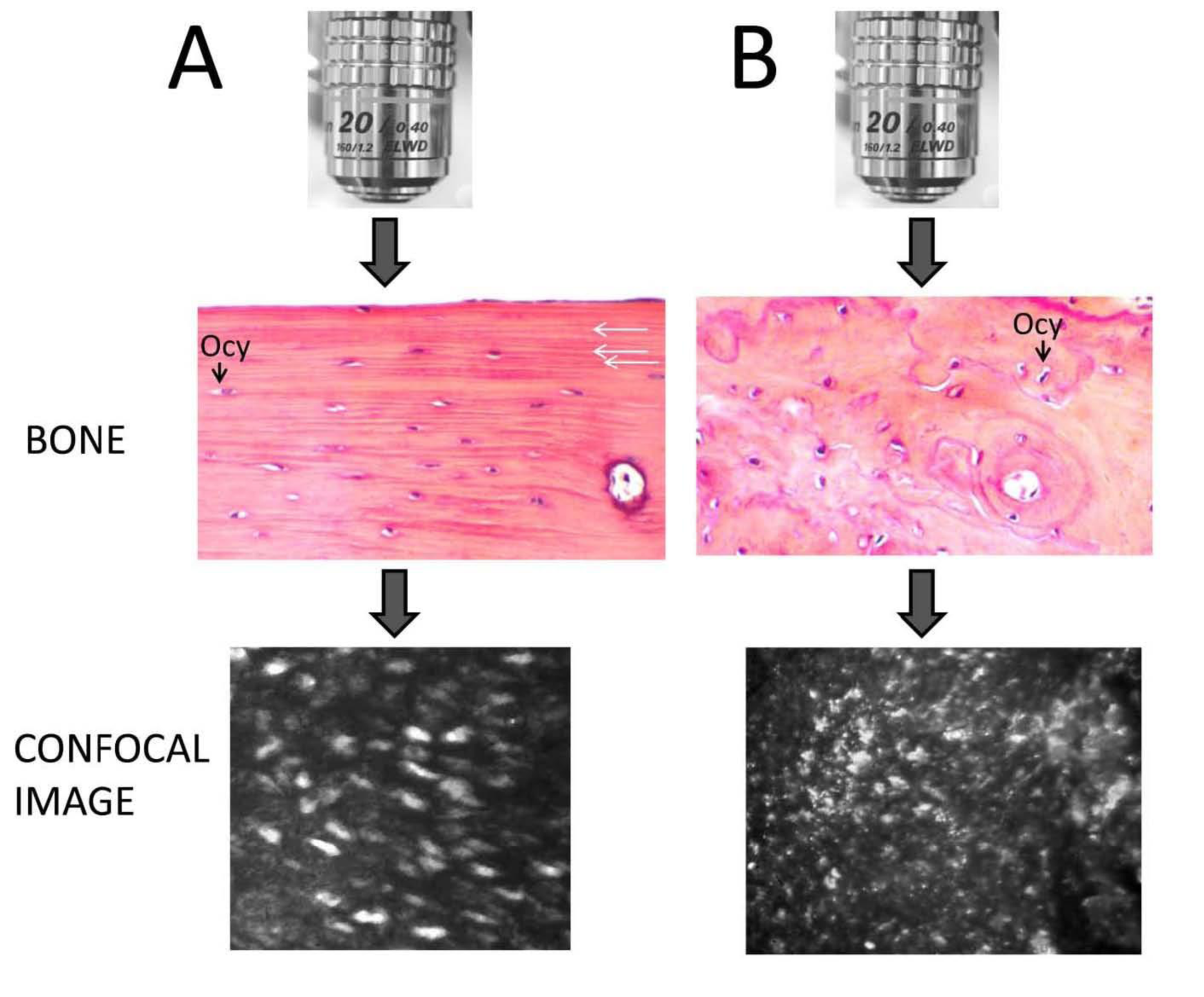

Supplement: Figure S1 — Identification of osteocyte orientation and bone forming characteristics. When periosteal surfaces are forming, bone is typically laid down as a parallel-fibered tissue or arranged in sheet-like structures called lamellae. Bromage and Boyde (2008) [7] described that osteocytes within these tissues are spatially well organized and have their long axes preferentially orientated with the principal orientation of the bone collagen. In contrast, osteocytes beneath resorbing periosteal surfaces are not typically well organized. To illustrate this principle, we have analyzed histological bone section of rat mandibles. Animal and Tissue Preparation: All animal manipulation conformed to University and Federal Guidelines. Two ∼100 g rats were perfused intracardiacally with 4% paraformaldehyde (PFA), mandibles were dissected free and soft tissues removed. After additional overnight fixation of the mandibles in PFA, samples were decalcified for 3 weeks in 4% EDTA, washed in buffer, embedded in paraffin and sectioned. Sections were stained following standard Hematoxylin-Eosin protocols and cover slipped to be imaged by an Olympus BH2 microscope. Figure S1, Panel A, shows paralleled fibered lamellar bone tissue (small white arrows) and osteocytes (Ocy), the latter showing a predominant orientation following that of the lamellae. In contrast, Panel B shows poorly identifiable lamellae and osteocyte orientation is random relative to the field of view. This principle applies to the imaging of ATD6-69 using portable confocal microscope as described [43]. The resulting confocal images for bone resorption or deposition are shown at the bottom of Fig. 1S. The confocal image on the bottom of Panel A thus represents forming bone surfaces with osteocytes arranged in a paralleled fashion. This image was taken from ref [7]. The image at bottom of Panel B was obtained from the clivus area of ADT6-69 and shows haphazard orientation of osteocytes thus representing a resorptive surface. In both [file pone.0065199.s001.tif]

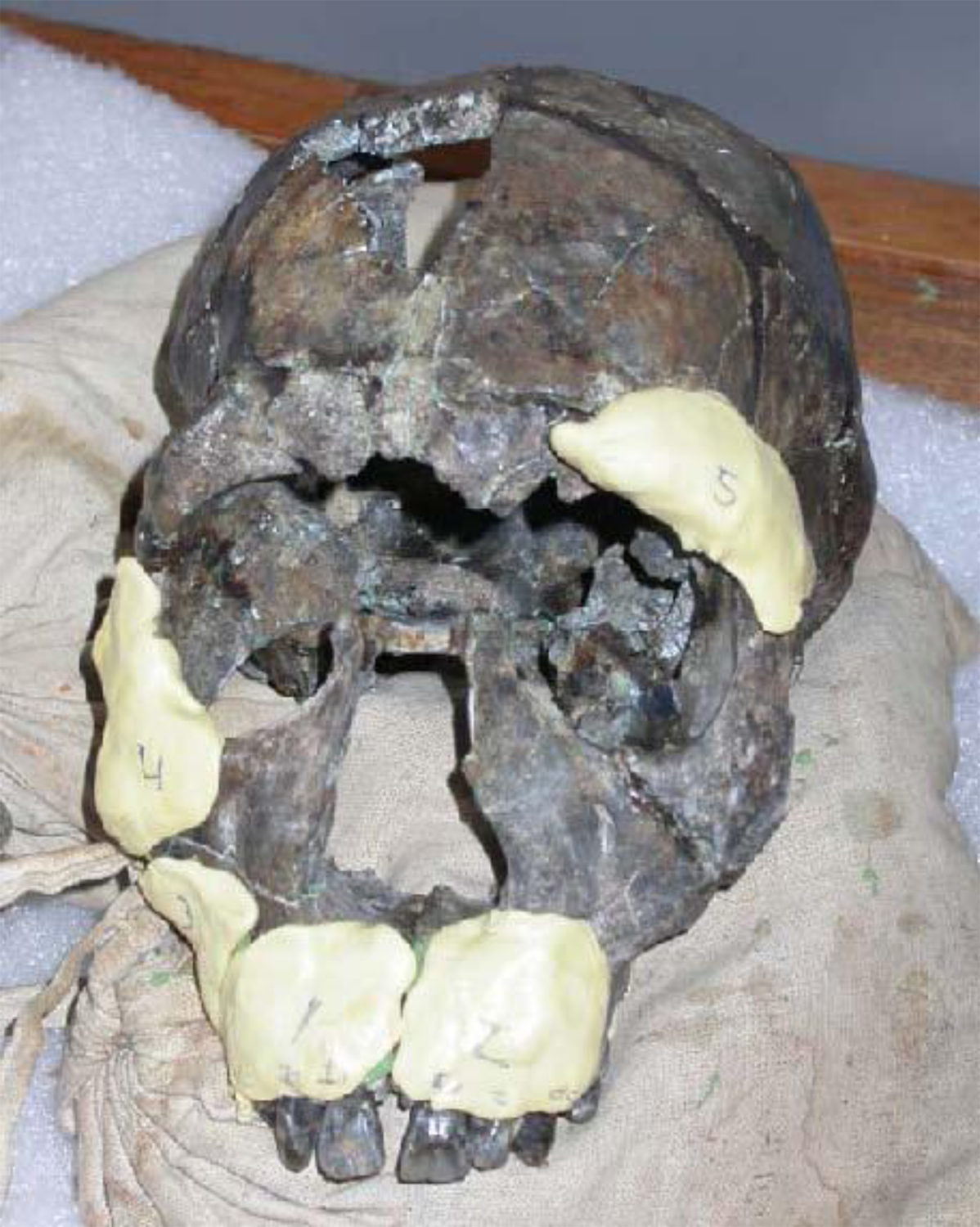

Supplement: Figure S2 — KNM-WT 1500 during casting of key areas of the face. KNM-WT 15000 during casting of key areas of the face. The facial skeleton of the Nariokotome boy specimen (KNM-WT 15000) was carefully prepared for study onsite at the National Museums of Kenya by one of us (RSL) by removing adherents. This procedure was carried out whilst periodically examining the specimen under a dissecting microscope to ensure that preservatives had been completely removed without damage to the specimen. Only selected areas of the specimen were cleaned. Areas where bone was thin or seemingly fragile and surfaces near glued areas were avoided. Small patches of the face and mandible were replicated as described above, labeled, and photographs taken for record keeping while the impression materials was still in place. Permission to study the specimen was kindly granted by the authorities of the National Museums of Kenya and supervised by Dr. Emma Mbua. (TIF) [file pone.0065199.s002.tif]
